# Statistical Methods for Degradation Estimation and Anomaly Detection in Photovoltaic Plants

**DOI:** 10.3390/s21113733

**Published:** 2021-05-27

**Authors:** Vesna Dimitrievska, Federico Pittino, Wolfgang Muehleisen, Nicole Diewald, Markus Hilweg, Andràs Montvay, Christina Hirschl

**Affiliations:** 1 SAL Silicon Austria Labs GmbH, Europastr. 12, 9524 Villach, Austria; vesna.dimitrievska@silicon-austria.com (V.D.); wolfgang.muehleisen@silicon-austria.com (W.M.); christina.hirschl@silicon-austria.com (C.H.); 2 Fronius International GmbH, Guenter Fronius Straße 1, 4600 Thalheim bei Wels, Austria; Diewald.Nicole@fronius.com; 3 ENcome Energy Performance GmbH, Lakeside B08b, 9020 Klagenfurt, Austria; markus.hilweg@en-come.com; 4 SAL Silicon Austria Labs GmbH, Inffeldgasse 33, 8010 Graz, Austria; andras.montvay@silicon-austria.com

**Keywords:** PV system, failure detection, degradation analysis, machine learning, operation & maintenance

## Abstract

Photovoltaic (PV) plants typically suffer from a significant degradation in performance over time due to multiple factors. Operation and maintenance systems aim at increasing the efficiency and profitability of PV plants by analyzing the monitoring data and by applying data-driven methods for assessing the causes of such performance degradation. Two main classes of degradation exist, being it either gradual or a sudden anomaly in the PV system. This has motivated our work to develop and implement statistical methods that can reliably and accurately detect the performance issues in a cost-effective manner. In this paper, we introduce different approaches for both gradual degradation assessment and anomaly detection. Depending on the data available in the PV plant monitoring system, the appropriate method for each degradation class can be selected. The performance of the introduced methods is demonstrated on data from three different PV plants located in Slovenia and Italy monitored for several years. Our work has led us to conclude that the introduced approaches can contribute to the prompt and accurate identification of both gradual degradation and sudden anomalies in PV plants.

## 1. Introduction

Evaluating the status of a PV plant is an important task in maintaining a high output performance and low operating costs. Operation and maintenance (O & M) companies aim at detecting any failure in a PV system and taking suitable countermeasures. Considering the cost-effectiveness of the different techniques for failure identification (visual inspection, thermography, electroluminescence, etc.) an efficient procedure for a plant evaluation is to first check for any power loss recorded by the monitoring system, followed, if needed, by other on-site techniques for identifying the plant failure [1]. Efficient and reliable methods, appropriate for online monitoring, should be used to detect any failure that causes power losses. A power loss in a PV plant can be correlated to the values of current, voltage, temperature, irradiance, thermal cycling, shading, and others [2]. While shading is difficult to measure and quantify, the other parameters can be measured within the PV plant monitoring system. Failures in a PV plant can be located in the PV modules, inverters, cables and interconnectors, mounting, or other components. Typical failures [1,3] located in PV modules include cracks, potential induced degradation (PID), burned marks and hail damage of the cells, soiling or physical damage as failure of the front glass, delamination as failure of the encapsulant, and others. Because of the many different types of failures, identifying one type of failure in a PV system is a challenging task. Nowadays increasingly more research is being done on diagnosing a specific set of failures [4,5,6,7].

A PV performance analysis involves the estimation of the long-term degradation rates, that quantify the gradual reduction of performance of a PV system over time. In many cases the degradation rates are calculated based on a metric called the performance ratio (PR) [8,9,10], which is the ratio of the measured and nominal power. Variants of the standard PR include a corrected PR that uses a corrected measured power to compensate for the differences in measured irradiance and module temperature, with respect to the Standard Test Conditions (STC). For example, a corrected PR is used in [9,11,12]. PR can be calculated on a yearly, monthly, or daily basis, after which an analysis of the PR time-series is done to evaluate the degradation. When a linear degradation over time is assumed, methods based on linear regression models and seasonal decomposition have been mostly used [13]. A simple linear regression model fits a linear model to the raw PR time-series [10], or to the trend component extracted after seasonal decomposition [8]. In another approach, the degradation ratio is extracted from the distribution of the year-on-year degradation calculated as the rate of change of the PR between the same days in two subsequent years [9]. For cases of nonlinear degradation rates, change point analysis has been performed to detect the changes in the degradation slopes, after which linear degradation rates are calculated between every two consecutive points of change [14].

Appropriate preprocessing and filtering of the dataset is needed to eliminate outliers, noise, and minimize seasonal oscillations [11]. An investigation of the uncertainty of several different methods for degradation estimations shows that the simple linear regression performed on the PR time-series has higher uncertainty than the methods that use seasonal decomposition [8,11]. However, an important requirement for the seasonal decomposition methods is an accurate estimation of the model parameters [11]. On the other hand, using a corrected PR requires a valid measurement of irradiance and module temperature, which in some cases are not available in a monitoring system. There is then the need of defining statistical approaches for degradation estimation that can either be used without the environmental sensor data, or that are not dependent on the accuracy of the seasonal decomposition models.

Besides gradual degradation, the performance of a PV plant can undergo sudden changes caused by localized failures in the system. A variety of statistical methods have been used for failure diagnostics, mostly involving machine learning (ML) regression models [13,15]. ML regression models have been used to monitor the operation of a PV system by estimating the expected output, being it either power, current, or voltage, and identifying as anomalies all instances where the measured output deviates from the predicted one. One approach to estimate the expected power output involves deriving the parameters of the standard nonlinear models of the relationship between current and voltage values, which are usually given by the PV module manufacturer, but are not always available [16]. Other approaches predict an expected daily power output, taking as input a combination of environmental data and data specific to the PV plant [17,18]. For this purpose, ANN (Artificial Neural Network) [18], SVM (Support vector machine) [17] and Regression trees have been used as regression models. Some results show a great performance of the ML models, obtaining a high correlation of more than 0.99 between the measured and predicted power output [18]. For a real-time optimum voltage and current prediction, recurrent ANN are investigated in [4], showing high accuracy of more than 98.2% [4]. An alternative approach of using data-driven models for power prediction is to use the one-diode model [19,20]. A comparison between the performance of the one-diode model and a recursive linear regression model showed a better performance in the regression model [6]. Once a regression model is derived, this can be used by failure detection algorithms. In some studies [16,20], to perform the fault detection, both the measured and predicted outputs are used. In one case, upper and lower boundaries of the loss in power are set up in advance based on which a fault is detected [20]. In another study, a weighted moving average control chart of the power residuals is used [16]. However, all these methods for output regression and fault detection have been so far tested only on one PV plant. For this reason, finding an approach for robust anomaly detection that can be used on several PV systems is still a great challenge as different systems may present different features.

The purpose of this work is to develop models for the assessment of the condition of a PV plant by monitoring the variation of its output. Two different sources for a decrease in performance are considered, i.e., progressive degradation and sudden anomalies. For each of these scenarios, multiple approaches for the plant’s assessment are considered and compared. For the detection of progressive degradation, we propose novel methods for degradation estimation that overcome some of the issues of the existing methods. More precisely, one of the methods does not rely on any environmental sensor data, and therefore it can be used in scenarios where these data are not available. The other method targets to find a reliable degradation evaluation without the use of any seasonal decomposition models, thereby avoiding the problem of an accurate estimation of the model parameters. For anomaly detection, instead, the developed approaches are based on regression models that predict the expected output for each inverter of the PV plant. We propose novel approaches to detect the anomalies by using the produced output. Compared to other approaches, our approach uses some of the measured data as training data. All the approaches considered in this work rely on statistical machine learning techniques and are therefore designed to be derived only from the available data without the need for an in-depth inspection of the plant. The proposed approaches are then validated on the data extracted from three different PV plants located in Europe, ranging from 4 to 19 inverters per plant, and each monitored for 5–6 years.

More in detail, Section 2.1 and Section 2.2 present the approaches developed for the estimation of the plant’s degradation and for the identification of anomalies, respectively. Section 3.1 and Section 3.2 discuss the applications of these methods to the selected PV plants, comparing the results. Finally, Section 4 draws the conclusions.

## 2. Methods

In the typical operation of a PV plant, two types of events can cause a decrease in performance:

- the progressive degradation of the plant due to aging, soiling, PID, or other degradation sources;
- sudden faults, that can affect a part or the entire plant, and are due to anomalous events, for instance failures or components breakdowns.

Both these types of events need to be properly monitored and recognized, in order for the plant to operate at its maximum efficiency. Because of the very different physical natures of these events, however, the methods that can be used for their assessment are necessarily different from each other.

### 2.1. Degradation Estimation

In order to obtain an accurate estimate for the plant’s degradation, one needs to monitor in time the value of some quantity that is supposed to remain constant in an ideal scenario, typically the plant’s power output. However, the challenge with monitoring a PV plant is that its output is continuously changing, because of the varying environmental conditions (temperature, irradiation, shadings, etc.). In this section, we then present two approaches for the derivation of a stable measurement for the plant’s output: one based on sampled values and the other on the prediction of the plant’s power in reference conditions.

#### 2.1.1. Sampled Values—Based Score

In [21] we defined an intuitive and computationally simple metric, called yearly degradation score (YDS), that quantifies the degradation in a PV plant between two or more consecutive years. One special characteristic is that it can be calculated, not only for the output power but also for several other data sources, including Maximum Power Point voltage (MPP-voltage, voltage) and Maximum Power Point current (MPP-current, current). That distinction could narrow down the failure types that could have caused the power loss in the plant. Similar ideas for differentiating between the degradation of the two components of the power (voltage and current) have been used in other methods for PV fault identification [16,20].

**Figure 1:**
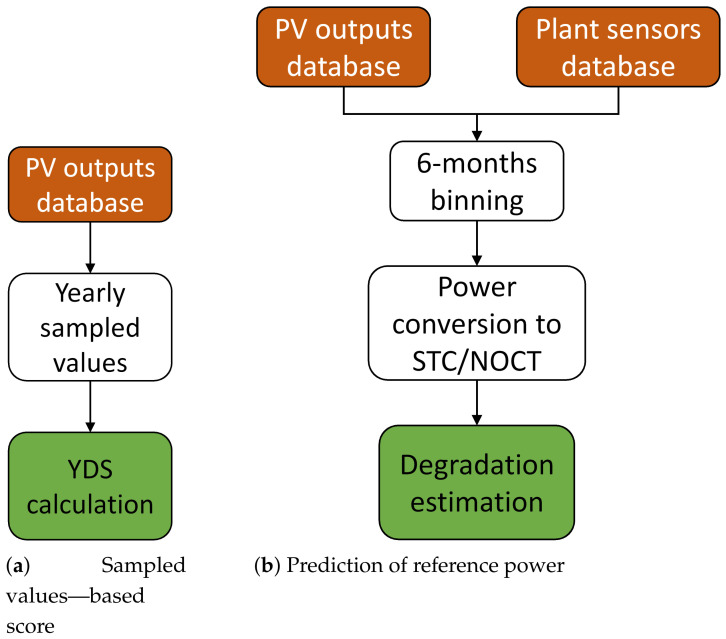
Flowcharts describing the two approaches for degradation estimation.

Instead of analyzing the whole data series, the idea behind YDS was to focus the analysis only on a representative set of raw values that give a nice reflection of the overall data. Taking this into consideration, YDS is calculated based on a selected set of K sampled values per year taken from the previously cleaned and filtered raw data. The highest values per year in voltage, current or power are selected in the representative set of values. The degradation score YDS is obtained from the slope of the linear line fitted to the selected points. Therefore, the slope represents the per unit reduction in the measured values per year. The final score of YDS is the percentage of degradation per year. The reference value used for the percentage calculation is the value of the fitted model in the first year. The whole flowchart of the method is shown in Figure 1a.

The performance of YDS depends highly on the preconditioning step where the data errors, data outliers, and data with unusual values are filtered out. The score is also affected by the value of the parameter K. A larger value of K could reduce the power of detecting smaller losses, while a smaller value could cause a greater influence by the outliers. The results showed that the best values of K are between 30 and 50 [21], so in the experiments done here K is set to 30. Note that the model presented here makes use only of a single time-variable input, being it either current, voltage, or power. Despite this, however, as will be shown in Section 3.1, the careful choice of input data can compensate the inherent variability, allowing to obtain a prediction performance very close to the one of much more complex models that make use of several input variables.

#### 2.1.2. Prediction of Reference Power

The previous approach relies only on the availability of the plant’s raw output data (power, current, and voltage). Most modern plants are, however, equipped with multiple sensors that can provide additional information over the operating conditions, most notably about the irradiance and the temperature. Using this additional information can then allow the derivation of a more robust model that can compensate for the variations in operating conditions during the year, with the added benefit of needing much shorter acquisitions to obtain enough data for the degradation estimation.

For these reasons, our second approach for the estimation of the PV plant’s degradation involves estimating the output power in conventional test conditions given the latest observed data. These test conditions are the common ones defined for PV modules [22,23] and are give as follow:

- Standard Test Conditions (STC): Irradiance = 1000 W/m^2^, Module temperature = 25 °C;
- Nominal Operating Cell Temperature (NOCT) conditions: Irradiance = 800 W/m^2^, Module temperature = 45 +/− 3 °C, Ambient temperature = 20 °C;

The STC are the conditions that correspond to the modules parameters communicated by the manufacturer, however, they are also difficult to realize in the real-world operation of the modules. On the other hand, the NOCT conditions are much more representative of normal operation.

The proposed approach consists of dividing the data in 6-month bins and deriving for each inverter a model that estimates the power at STC and NOCT conditions in each bin. The inputs to the model are the raw irradiance and module temperature for the STC, while in NOCT conditions also the ambient temperature is added. The chosen model is a Decision Tree [24], because of the high efficiency and ease of training and interpretation, and a different model is trained for each of the 6-month bins. The model is then trained to predict the expected power given any values of irradiance and temperature, and the trained model is finally used to estimate the power at STC and NOCT conditions. For better model accuracy, only the data points where the irradiance difference with respect to the test conditions is lower than 150 W/m^2^ and the temperature difference lower than 5 °C are considered. Due to the unrealistic STC, though, this results in much fewer data points available in this case with respect to NOCT conditions. All data are normalized to lie approximately in the interval [0,1] for better numerical properties, and the whole training procedure is handled in Python using the Scikit-Learn library [25]. The decision tree model uses as criterion the Friedman MSE, while the minimum number of samples to create a tree leaf is set to 10. The whole flowchart of the method is shown in Figure 1b.

### 2.2. Anomaly Detection

While a progressive degradation of a PV plant’s performance is inevitable due to the aging of its components, other events can cause a sharp decrease in power output, and therefore need to be promptly identified and corrected. As these events are usually very localized in time, the approaches presented in Section 2.1 are not appropriate because they require the collection of data over long time periods, spanning at least a few months. On the other hand, for a prompt fault detection, there is the need of developing methods that can immediately signal if any anomaly is occurring. In this section we present two approaches, one based on the real-time prediction of the inverter’s DC currents and voltage from environmental information and the other monitoring the deviations of each inverter from the behavior of a reference inverter.

#### 2.2.1. Environmental Model and Control Chart

One way of detecting an anomaly is to build a prediction model for the instantaneous DC current and voltage outputs of each inverter, given the current conditions in terms of irradiance and temperature. This approach shares many similarities with the one presented in Section 2.1.2, however, here, we do not try to predict the power in reference conditions, but rather the instantaneous values of DC current and voltage at each time step at the current environmental conditions. Such a model needs to be trained on a dataset that summarizes the behavior of the inverter in normal operating conditions. The anomaly detection is performed by comparing the measured current and voltage to the predicted ones making use of control charts [26].

In this work we have chosen again Decision Trees for the models, as in Section 2.1.2 and using the same normalization strategy and implementation details. The prediction task involves estimating separately the DC current and voltage at each time-step for each inverter. The inputs to the model are the measured irradiance and ambient temperature at the same time-step where the prediction is calculated. As the module temperature is not always available in every plant (for instance plant B in Section 3), we have decided not to use this measurement in the model.

**Figure 2:**
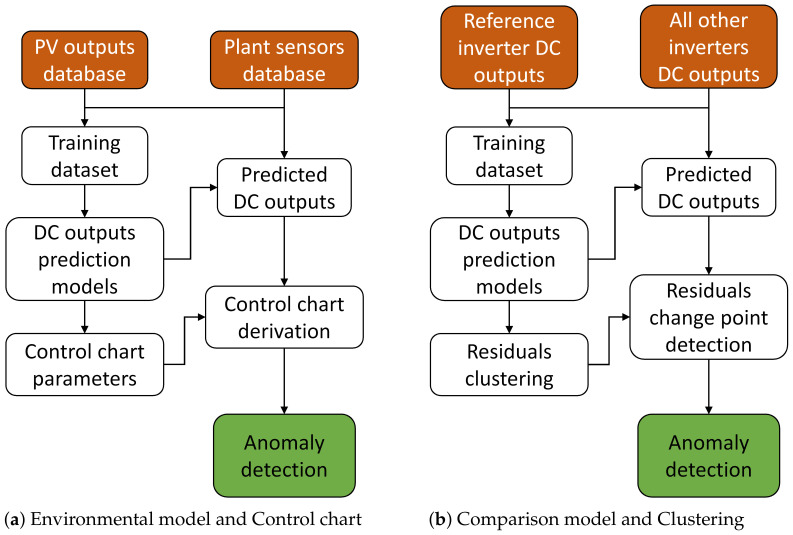
Flowcharts describing the two approaches for anomaly detection.

Having derived a model on the training set, for the derivation of the control chart the residuals need to be calculated. To compensate for the daily variability, the residuals *r* are aggregated per day *D*:

(1) $$r^{D}=\frac{\sum_{t\in D}\frac{1}{N^{D}}\left|X_{pred}^{t}-X_{meas}^{t}\right|}{\max_{t\in D}X_{meas}^{t}}$$

where *t* indexes the measurement samples, $N^{D}$ is the number of samples in day *D* and $X_{pred}$ and $X_{meas}$ are either the predicted or measured, respectively, current or voltage. These residuals are used for the derivation of the control chart, which identifies as anomaly all points in which:

(2) $$r^{D}>r_{0}+3\sigma_{r}$$

where $r_{0}$ is the average and $\sigma_{r}$ the standard deviation of $r^{D}$ on the training set. The whole flowchart of the method is shown in Figure 2a.

#### 2.2.2. Comparison Model and Clustering

The disadvantage of the previous approach is that it requires identifying, for each inverter, a pool of data that is considered “normal operation” for the training of the models. These data have to include not only the output of each inverter at every time-step (power, current, and voltage), but also the environmental information (irradiance, and temperature), which is not available in every plant. Moreover, these models need to be periodically retrained to account for the gradual degradation due to aging, as discussed in Section 2.1.

For these reasons, we developed a second approach for anomaly detection that aims at detecting unusual daily patterns by comparing the deviations with respect to a reference condition. In order to account for the great seasonality effect and dependence on the weather conditions, the approach presented here compares the operations of a chosen inverter, called reference inverter, to all other inverters. The comparison is done based on DC current and DC voltage. For this purpose, a statistical ML model predicts the value of one inverter, given the value of the reference inverter. The advantage of this approach is that it does not require environmental information and that, assuming the whole plant ages uniformly, the models do not need to be retrained periodically. It does, however, assume that the train data, based on which the prediction ML model is created, is representative of normal operation, without any anomaly.

After the prediction model is created, the daily residuals are calculated as the difference between the modeled and measured values. In the next step, clustering is performed on the daily residuals in the train data. A K-means clustering algorithm specialized for time series data is used for this purpose. The Python package “tslearn” [27] is used for the implementation. The distance metric “Dynamic time Warping”(DTW) [28] is selected for clustering as it can be used to calculate the distance between time series with different lengths. The general idea for calculating the DTW distance is to find the one-to-many and many-to-one matches that will minimize the total distance between the two time series. As a result, small shifts in relation to time should not affect the DTW distance, and even short-term missing data or outliers should have a smaller effect on the metric. In order to find the best fitted number of clusters for the train data, an iterative search between 2 and $N_{max}$ is performed, where $N_{max}$ is the maximal number of clusters. The best fitted model is the one with the highest "Silhouette Coefficient" (SC). The parameter $sih_{min}$ is defined as the minimal value of the SC that an accepted cluster model should have. Therefore, if the best model has a SC less then $sih_{min}$, then the best model is set to the one with only one cluster. The clusters found with the best model in the train data are further inspected. All clusters with less than $count_{min}$ items are discarded as invalided clusters, where $count_{min}$ is the minimal number of items allowed in one cluster.

To detect the unusual daily residuals in the test data that do not fit into the clusters of daily residual in the train data, one unsupervised change point detection algorithm is used [29]. We used a variant of the “Model Fitting”(MF) event detection algorithm [30]. According to the original MF algorithm, a change point is detected in a time series if the Euclidean distance between the point and all clusters found in the time series is higher than the radius in each of the clusters. In our implementation, the radius of a cluster *C* ($r_{C}$) is the maximal DTW distance between all items in the cluster and the center of the cluster ($\mu_{C}$) calculated by the clustering algorithm. Therefore, if the DTW distance between a daily residual and the center of each cluster is higher than its radius, an unusual daily pattern is found.

To be able to quantify how a daily residual *x* differs from the clusters, a distance $d_{x}$ is calculated using the formula in Equation (3), where $C_{set}$ is the set of all clusters found and $d_{x,C}$ is the distance to the cluster *C*. If *x* is fitted into the cluster *C*, the distance is 0, while otherwise a relative DTW distance limited to 100% is obtained. The whole flowchart of the method is shown in Figure 2b.

(3) $$d_{x}=\min_{C\in C_{set}}d_{x,C}=\min_{C\in C_{set}}\left\{\begin{array}{c} 0,\mathrm{if}\;x\in C\\ \min(\frac{DTW(\mu_{C},x)}{r_{C}}100\%,100\%),\;\mathrm{otherwise} \end{array}\right.$$

## 3. Results

For our investigation we have made use of data coming from three plants. A crystalline silicon technology is used in all plants. For anonymity reasons, we will call them plants A, B, C, and they have the following characteristics:

- plant A, location Slovenia: data acquired between September 2013 and April 2020, however with some long interruptions due to lack of data from some sensors, 19 inverters have been considered. In this plant, global irradiation, and ambient and module temperatures are measured. The irradiation sensor is based on a thin film solar cell, whereas the module temperature sensor is a Pt100. The installed capacity of the plant is approximately 315 kWp and contains 1313 pieces of 240 Wp modules composed of multicrystalline silicon (multi-c-Si) cells. Regarding the placement of the modules, there are two different orientations: southwest (200°) and southeast (135°). More precisely, there are 12 inverters with southwest orientation, 6 have southeast orientation, while one inverter has modules connected to both orientations. Moreover, the irradiation sensor shares the same southwest orientation of the first 12 inverters.
- plant B, location Sardinia, Italy: data acquired between April 2014 and April 2019, 4 inverters have been considered. Around 4/5 of the modules are west-oriented and the others have southwest orientation. This plant has crystalline silicon (c-Si) cells. The measurement sensors consist of an irradiation and ambient temperature sensor. The irradiation sensor is based on a silicon solar cell, while the module temperature is not measured.
- plant C, location Italy: data acquired between January 2011 and April 2020, 5 inverters have been considered. Each inverter is connected to three strings, each with 16 PV modules. All inverters have modules with the south orientation. In this plant, irradiation, ambient and module temperatures are measured. The plant is composed of crystalline silicon technology modules. No specifications of the sensor technology is available.

The investigated plants have very different sensor infrastructures and do not always have detailed information about the sensors available. For these reasons, the focus of this paper is not on the condition of the sensors, which we investigated in [31], but rather on the methods for deriving reliable prediction models using a variety of possibly unknown sensors. It can also be observed that, in plant A, the modules and the irradiance sensor come from different technologies. According to the work in [32], using amorphous silicon irradiance sensors, which are a much cheaper technology, in a c-Si plant is not optimal, but this should result only in a fixed offset. However, as the prediction models presented in this work learn the relationship between the irradiance values and DC plant values from the measured data, such an offset is automatically compensated.

Moreover, for some of the plants the status of the investigated strings has been assessed with an on-site inspection. For plant A the inspection using thermal imaging showed inactive parts in the PV modules, that explains the higher degradation in voltage which will be shown in Section 3.1. For plant B, no on-site inspection could be performed. Finally, for plant C, the on-site inspection using thermal imaging and IV-curve measurements showed only a slight PID behavior, but an overall good operation of the plant with no suspicious behaviors, consistently with the results in Section 3.1.

### 3.1. Degradation Estimation

A well-operating PV plant using the crystalline silicon technology has an estimated power degradation due to aging of 0.5–0.6% per year [33]. The estimated degradation of the inspected plants, using the proposed approaches, are presented next. These results are also compared to a popular method where the degradation is calculated based on a linear standard least square regression applied to the temperature corrected PR [10]. The data are first filtered using appropriate irradiance, outlier and stability filters as suggested in [10,12]. The reference PR-based degradation rate is calculated only for plants A and C, for which the module temperature and the coefficients needed to calculate the PR are known. Because of the high difference in these datasets, the applied outlier filter was customised for both datasets.

#### 3.1.1. Sampled Values—Based Score

**Figure 3:**
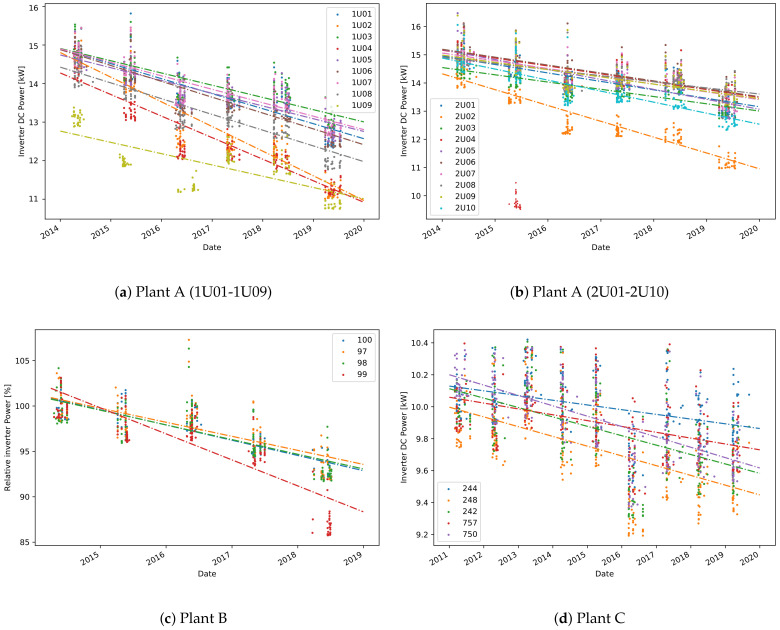
DC power degradation for all inverters in the plants calculated using the sampled values-based approach.

The estimation of the plant’s degradation using the sampled values-based method on the DC power is performed for all plants, Figure 3 and Table 1 and Table 2. For each inverter, the sampled values taken for each year are shown in the plots, together with a linear fit showing the degradation. In Figure 3c, the relative values in percentage are given for better clarity since the range of power values for inverters 97 and 98 is about 5 times higher than the one of the other inverters. For the other plots in Figure 3, the absolute values expressed in Watts are given. The yearly degradation of the DC power for all inverters in plant C is on average 50 W per year, or 0.5% per year. Plants A and B have higher degradation. More precisely, on average there is a yearly degradation of 1.9% for plant B and 2.5% for plant A.

By comparing the degradation rates per inverter in one plant, interesting results can be observed. First, inverter 99 (Figure 3c) has a higher degradation in power than the other inverters. Next, there is an unusual drop in the sampled values in 2016 shown in Figure 3d, that has no significant effect on the linear degradation fit. Finally, there is a higher degradation in power for some of the inverters in plant A, such as 1U02, 1U04, 1U09, and 2U02 (Figure 3a,b). A significant drop in DC power is seen for the inverter 2U04 in 2015, where the selected points deviate highly from all others. Including these sampled points in the degradation analysis affects highly the YDS. Hence, for better accuracy, these selected points are omitted from the degradation analysis.

**Figure 4:**
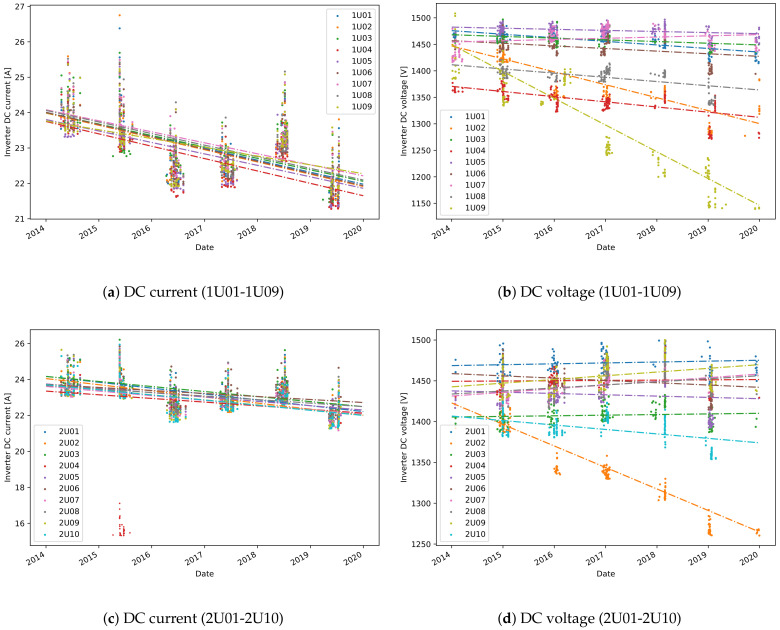
Estimated degradation in DC current and DC voltage for plant A.

One valuable feature of the sampled value-based approach is that the degradation in DC current and DC voltage can also be obtained. Consequently, the power degradation can be correlated to the degradation in DC current or DC voltage. One can observe that the higher degradation in power for some of the inverters in plant A, is related to a higher degradation in voltage (Figure 4). A detailed comparison of the degradation rates, expressed in percentage, is shown in Table 3. Although, theoretically a loss in voltage is not expected, for several inverters, like 1U02, 1U09 and 2U02, there is loss in voltage of above 1% per year. The average percentage of degradation in DC current for all inverters is 1.2%, but there is no significant difference between the degradation of different inverters. One explanation of such uniform degradation between the inverters is that it is a result of accelerated aging or soiling.

Similar analyses on the other datasets bring additional observations. First, the degradation in power for inverter 99 in plant B, is related to a loss in current. Next, the slight power degradation in a few of the inverters in plant C can be correlated to a higher degradation in DC voltage (Figure 3d, Table 2). Lower selected values for DC voltage of approximately 460 V is seen in the period 2015-2017, compared to 2011 when the values are approximately 480 V. The obtained degradation rates are in high correlation with the referenced degradation method based on PR (Table 2). The advantage of using the sampling method is that it does not depend on either the temperature coefficient for the power or the nominal power used for a reliable PR calculation. During the on-site inspection of this system, it was found that a slight PID effect is distributed across the system, which has different effects on the various inverters.

#### 3.1.2. Prediction of Reference Power

**Figure 5:**
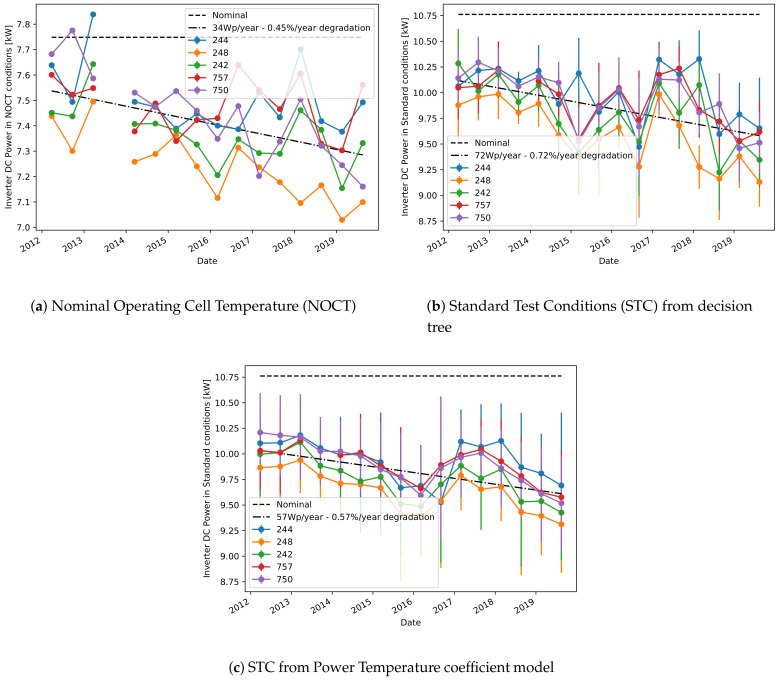
Predicted reference power for plant C.

The second approach for estimating the plant’s degradation (Section 2.1.2) has been applied instead only on plants A and C, due to the lack of the measured module temperature in plant B. Figure 5 shows the predicted power in STC or NOCT conditions for each of the considered inverters in plant C. All curves are collectively fitted with a linear model, to have an estimate of the overall decreasing trend. Moreover, the dashed horizontal line shows the nominal power as communicated by the modules manufacturer. The vertical lines at each point estimate the uncertainty of the power estimates, and this is always much higher in STC because of the lack of one prediction input (the ambient temperature) and the lower amount of data available for training the models. Additionally, Figure 5c shows the prediction of the STC power obtained using the standard Power Temperature coefficient model [34] for the dependency of current and voltage with irradiance and temperature, which makes use of the coefficients communicated by the modules manufacturer. Also in this case, the results are in accordance with the previous estimates of power and degradation based on the decision tree model, therefore validating our approach. However, the uncertainty indicated by the error bars is even higher in Figure 5b than the one in Figure 5c, therefore showing how the Power Temperature coefficient model can provide good prediction results only on average, but it is not suitable for precise point-wise estimations.

For a more precise estimation of the degradation, Table 2 shows the linear fit for the degradation obtained separately for each inverter and each operating conditions. As already observed, usually the estimates obtained in STC and NOCT conditions are rather different between each other. However, as the power estimation for NOCT is more reliable (as seen by the smaller uncertainty), we believe that these should be the conditions to be preferred, and we will just consider this case in the remaining of this work. Note also that the degradation estimation obtained from NOCT conditions is in good agreement with the one obtained using the sampled values model.

**Figure 6:**
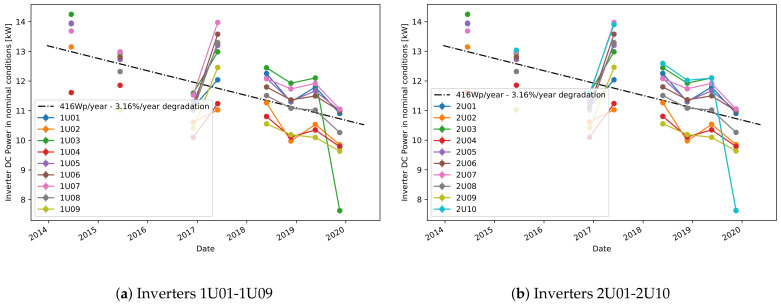
Predicted NOCT power for all inverters of plant A.

Moving then to plant A, Figure 6 shows the predicted NOCT powers for all considered inverters, together with their collective linear fit. Unfortunately, as immediately evident, the missing data prevents from obtaining continuous curves, however the plant’s operational time is still well covered. It can also be observed that the yearly degradation of this plant is much higher than in the previous case.

**Figure 7:**
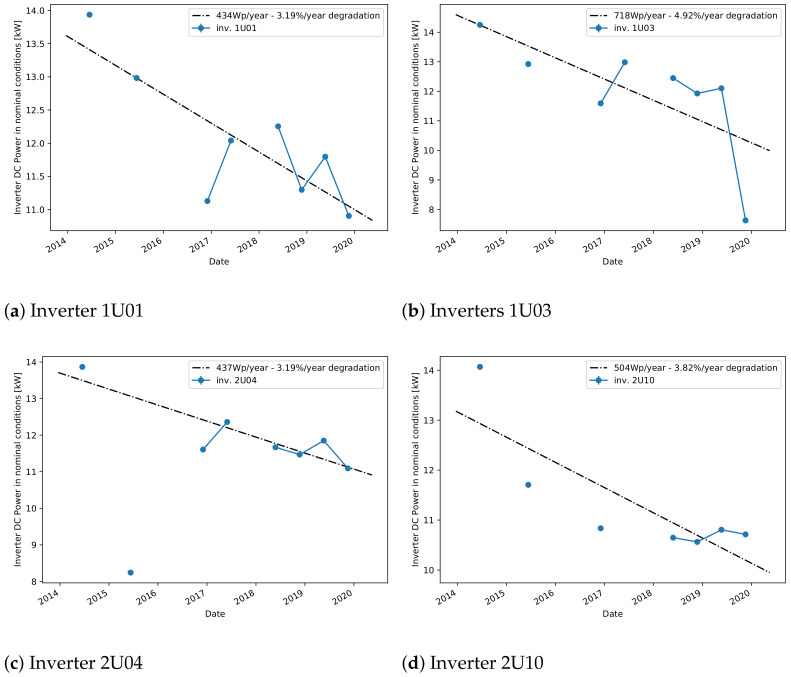
Predicted NOCT power for selected inverters of plant A.

A more detailed comparison is shown in Figure 7 where only four inverters are considered and individually fitted for linear degradation. The linear model is fitting the data very well, therefore reassuring about the validity of the proposed approach. The calculated yearly degradations are also relatively consistent between each other, showing that all these inverters are affected by the same phenomena. For a more detailed comparison, Table 1 also shows the calculated degradation coefficients for all considered inverters of this plant. Note again the good agreement between this model and the one based on sampled values. Comparing the degradation scores with the reference score obtained from the PR-based method, one can observe that the degradation ranges are within a similar range of around 1.5% to 3% per year. This high degradation rate can be explained by problems in the plant that were discovered during an on-site inspection. More precisely, disconnected cell failure [2] was found which was distributed throughout the system and affected all inverters, but each on a different scale. These findings were confirmed with thermal images. Although the PR method produces similar results, our methods are better adapted to typical data available for online monitoring where the information for the nominal power per inverter is normally absent or difficult to obtain. For instance, different inverters in plant A have different nominal powers that needs to be considered in the PR calculation, and this information might not be always available. Additionally, because the PR-based degradation rate was highly affected by the increasing trend present in the module temperature data from plant A, a recalculated module temperature obtained from the measured ambient temperature using a correction formula [35] was used instead. On the other hand, our method is less affected by the problems in the module temperature data because the model learns to predict the reference power from data blocks of 6-month data where this increasing trend does not have a high impact, resulting therefore in a more robust model.

### 3.2. Anomaly Detection

As discussed in Section 2.2, we have developed two approaches for anomaly detection, which both require as a first step the derivation of a regression model (Section 3.2.1). The anomaly detection algorithms are then developed and compared between each other (Section 3.2.2 and Section 3.2.3).

#### 3.2.1. Regression Models

##### Environmental Model

The first algorithm for the prediction of the inverters DC current and voltage uses the approach presented in Section 2.2.1. In this case only plants A and B are considered, and the recorded data have been divided in two parts: the first one, composed of all data acquired before the 1st January 2018, constitutes the training set for our models, while the second one, composed of all data acquired after this date, constitutes the test set on which the models performance is assessed.

**Figure 8:**
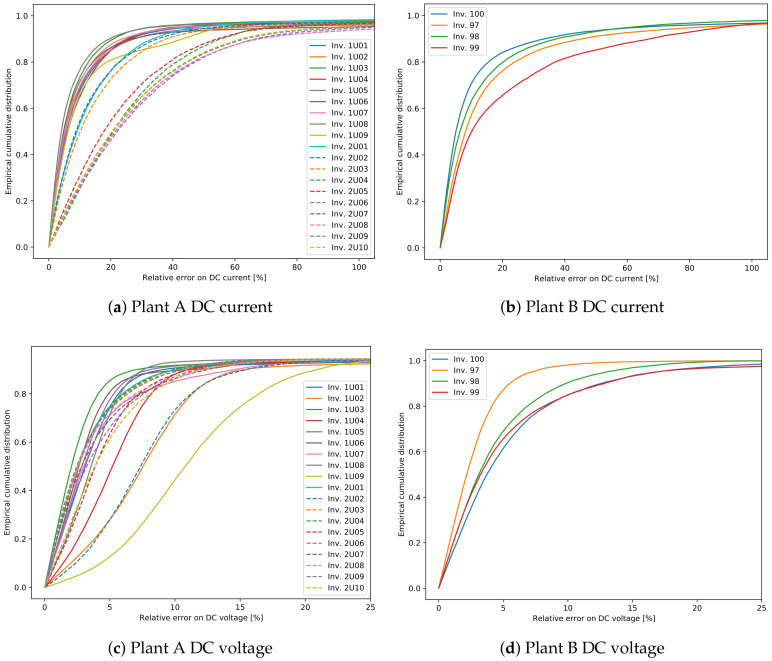
Empirical cumulative distributions of the relative errors in the predicted voltage and current for each of the two plants and each inverter on the test set. Only the points where the current is higher than 5% of the maximum measured inverter’s current are considered.

Figure 8 shows the cumulative distributions of the relative errors in the predicted voltage and current for each of the two plants and each inverter on the test set. For this plot only the points where the current is higher than 5% of the maximum measured inverter’s current are considered, in order to focus only on times of operation. As immediately apparent, the error on the voltage prediction is usually much lower than the one on the current, which is then the most important contribution to the error in predicted power. Noted that there is a relatively large difference in errors between the different inverters, which has to be investigated.

**Figure 9:**
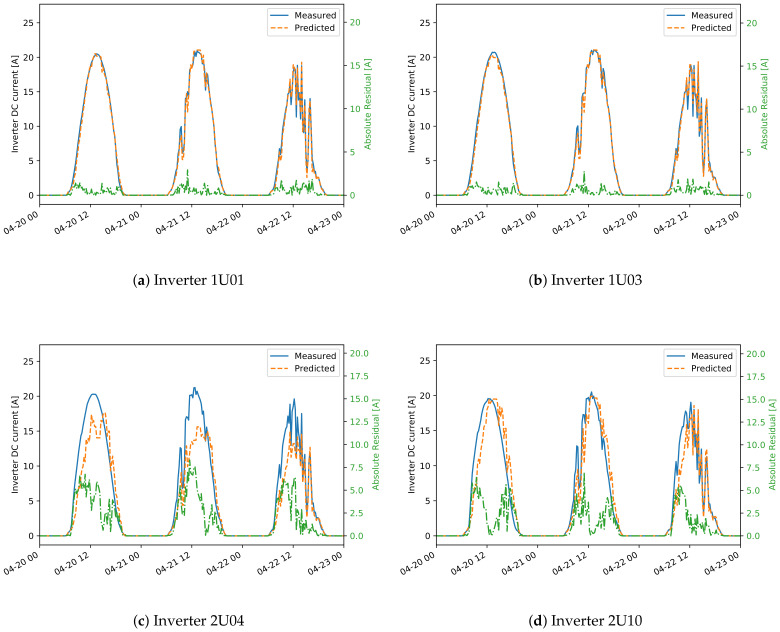
Detail for two inverters from plant A showing both the measured and predicted DC currents. The residuals are also shown (green dash-dotted line, right axis).

**Figure 10:**
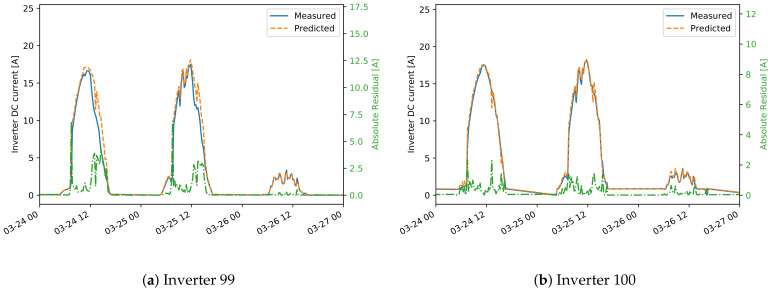
Detail for four inverters from plant B showing both the measured and predicted DC currents. The residuals are also shown (green dash-dotted line, right axis). The residuals are also shown (green dash-dotted line, right axis).

For this reason, Figure 9 and Figure 10 show comparisons between measured and predicted DC currents for some inverters of the two plants. For plant A (Figure 9), it is apparent that the inverters 1U01 and 1U03 have conserved the same behavior between the training and test sets, and for this reason the predicted current is always very close to the measured one. On the other hand inverters 2U04 and 2U10 have deviated much more from this behavior, exhibiting both a small shift in time, due to the slightly different orientation between these modules and the irradiance sensor, and higher measured current for inverter 2U04, probably due to improvements in the PV panels or in the inverter. Note that the time shift for inverters 2U04 and 2U10 is just a systematic error, which can in principle be compensated, but it does not affect the results of anomaly detection. This happens because the derivation of the control chart limits takes already into account and compensates for any systematic error.

For plant B (Figure 10), instead, the differences between the inverters is much smaller. It is, however, evident also in this case the time shift of inverter 99, which leads to a higher prediction error.

##### Comparison Model

The second approach for deriving a regression model of the inverter’s DC current and voltage involves the usage of a reference inverter (as explained in Section 2.2.2). The easiest method for choosing a reference inverter is to select any one that does not show any evident anomaly in the recorded data, and this is the choice made in this work. For an application of this method to online monitoring, however, methods for checking whether the reference inverter is still operating normally need to be implemented. Such methods can make use, for instance, of a second reference inverter that could promptly signal if any anomaly occurred on the reference inverters. Another possibility would be to monitor whether suddenly all inverters signal an anomaly at the same time, indicating a possible failure on the reference inverter. These investigations would however require data where the anomalies are precisely characterized, and are therefore left for future work.

In the case presented here, the reference inverters chosen for the plants are: 1U01 for plant A, 100 for B, and 244 for C. Approximately one year of data, taking the data from the start date of plant operation is used for training. The starting dates of the test data are the following: 1.1.2015 for plant A, 1.7.2015 for plant B, and 1.1.2012 for C. The implementation for the prediction models is done using the Scikit-Learn library [25]. The following algorithms were tested: “Linear regression”(LR), “Support vector regressor”(SVR), “Random forest regressor”(RFR), and “Decision tree”(DT). The parameters were set to their default values, except the maximal depth of the trees used in RFR that was set to 5, and the parameters for the DT models that were the same used in the approach in Section 2.2.1. For training, 70% of the data is randomly chosen, while the other 30% was used for evaluation of the prediction model. The evaluation showed that a simple LR model can predict the DC current with high performance, featuring a $r^{2}$ coefficient of around 0.97. On the other hand, $r^{2}$ is only 0.47 for the models that predict DC voltage, showing a much lower performance. This result was expected since there is a strong linear dependency between the irradiance and DC current that would cause the DC current of two different inverters to be linearly dependent. On the other hand, this is not valid for the DC voltage.

To overcome the limitations of linear models for DC voltage prediction models, experiments were conducted to evaluate the models SVR, RFR and DT. For a better performance, the input data for SVR were standardized, while for RFR and DT the data were normalized to [0,1]. Later, the reverse process was done to get the prediction in the same range as the measurements. Adding the temporal features: “Time in the day”, expressed in hours, and “Day in year”, expressed as the index of the date, is also evaluated. The average value of the root mean square error (RMSE) of the evaluation data, for all cases of models and input data, is shown in Table 4. Results suggest that the best performance is achieved when using the SVR model with the additional temporal data include in the input. Therefore, for further investigations, the models for DC voltage prediction make use of this method.

#### 3.2.2. Clustering

The daily residuals calculated from the comparison model, which employs a reference inverter (Section 2.2.2), are used to run the clustering algorithm and find the meaningful clusters in the training data. The daily residuals are represented as multidimensional vectors, where each dimension matches a time in the day when a measurement is done. Only the dimensions with enough valid data are considered in the vector. Before clustering, missing data in the beginning and in the end of the day are replaced by 0-values, while the other missing data points are interpolated from the surrounding values. Daily residuals vectors with more than two consecutive missing points are discarded for the training process. The parameters used in the clustering (Section 2.2.2) are set to $N_{max}=4$, $sih_{min}=0.5$, and $count_{min}=5$.

**Figure 11:**
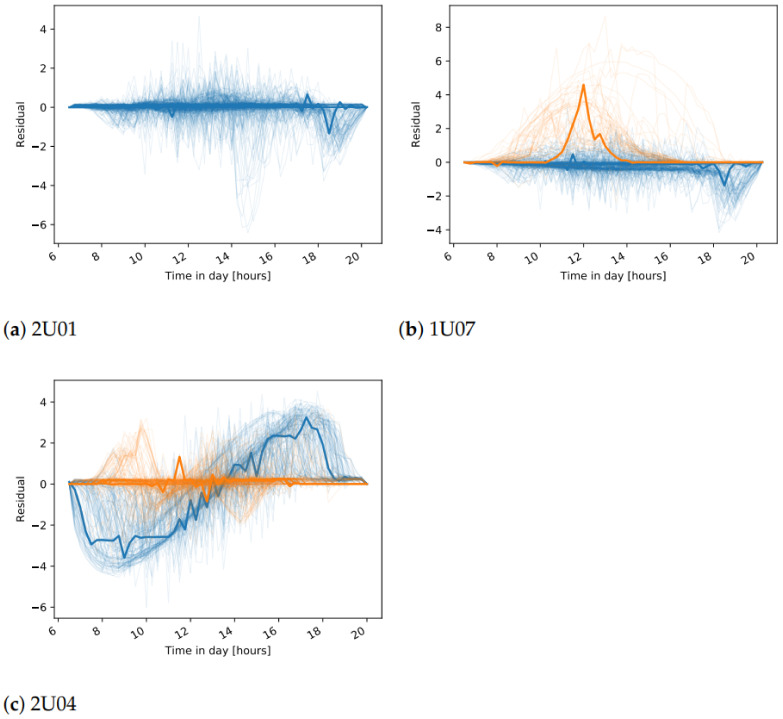
Clusters obtained from the daily residuals of DC current predictions for several inverters in plant A.

In many cases, as expected, only one meaningful cluster is found. In fact, if there are valid data from a well-operating plant, then the daily patterns of the residuals should be close to 0. Visualization of the daily residuals in a case where one cluster is identified, together with the cluster center, is seen in Figure 11a. If different states of operation are present in the training data, we expect that more clusters will show up. One such example is inverter 1U07, where erroneous data are present in the training set (Figure 11b), where two clusters are found. This is one drawback of the approach as there is no validation on the training data and we make an assumption that it does not include failures. Therefore, the final event detection for inverter 1U07 should be interpreted with caution. Another observation can be made for the inverters from plant A that have a different orientation than the reference inverter 1U01. For all inverters 2U04-2U10, two clusters are found in the DC current daily residuals, that are related to days in summer and winter seasons. The LR model cannot capture the shift in DC current seen for inverters with different orientation. Therefore, these shifts, that are different for the different seasons, are seen in the clusters (Figure 11c). The final observation is that in a few of the cases on DC voltage daily residuals, except for the expected cluster around the 0-residuals, an additional one is found. One explanation is that natural shadows cause the modeled and reference inverter to start and end the daily operation at different times of the day. Hence, higher residuals are seen at the start or end of the day, which is later identified as a separate cluster. It can then be concluded that such a second cluster also shows a normal pattern since it represents a particular feature of the inverter.

**Figure 12:**
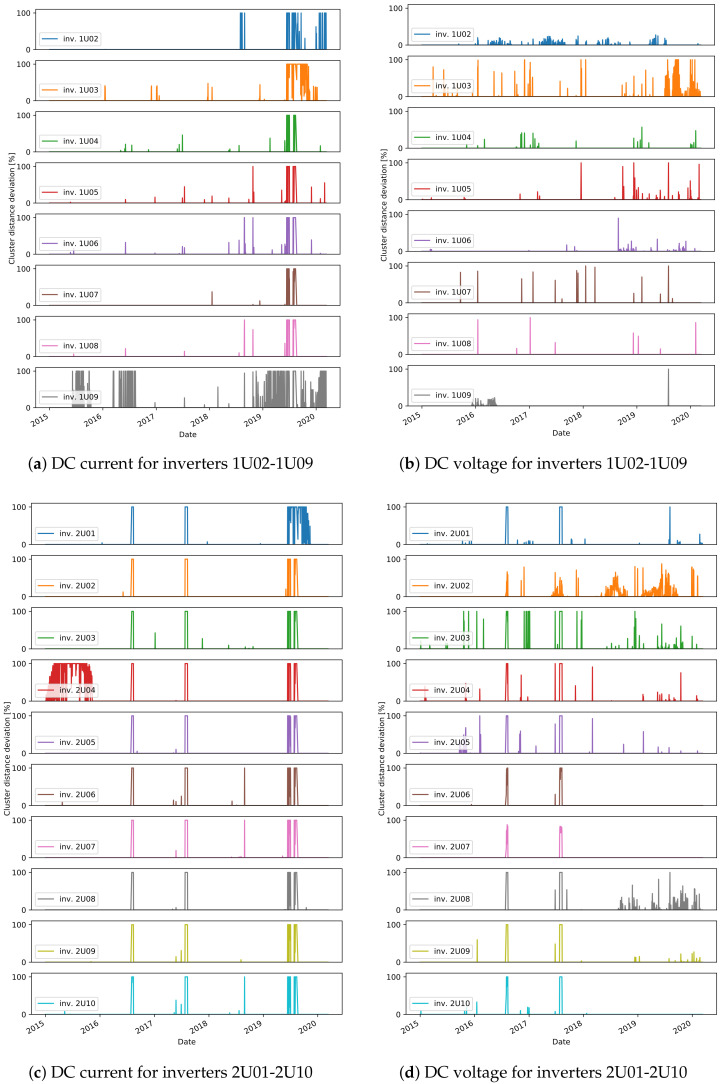
Unusual day event detection in plant A.

**Figure 13:**
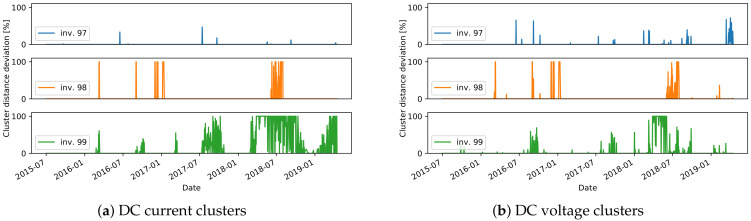
Unusual day event detection in plant B.

**Figure 14:**
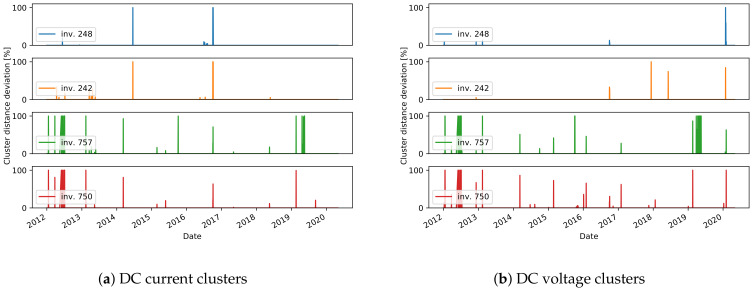
Unusual day event detection in plant C.

The final stage of the approach is to find the daily events that do not fit to any cluster. The relative distance of the daily residuals, for all days in the test data, are shown in Figure 12, Figure 13 and Figure 14. We consider the days with a distance higher than 0 to detect an unusual daily pattern. On average, for all inverters, 4% (4% ) of the days in plant A, 11% (7%) in plant B, and only 0.7% (0.7%) in plant C are identified as DC current (DC voltage) unusual events. With the distinctions between unusual events in the residuals of DC current and DC voltage, one can find failures connected to DC voltage or DC current issues.

As the ground truth information of failures in the systems is not available, the evaluation of the proposed approach to detect unusual daily events is done qualitatively. The investigation of the daily events suggests different scenarios:

1. one-day events specific to one inverter;
2. long-term events specific to one inverter;
3. events occurring on all inverters, indicating either a plant-wide failure or a problem on the reference inverter;
4. events detected on both DC current and DC voltage.

**Figure 15:**
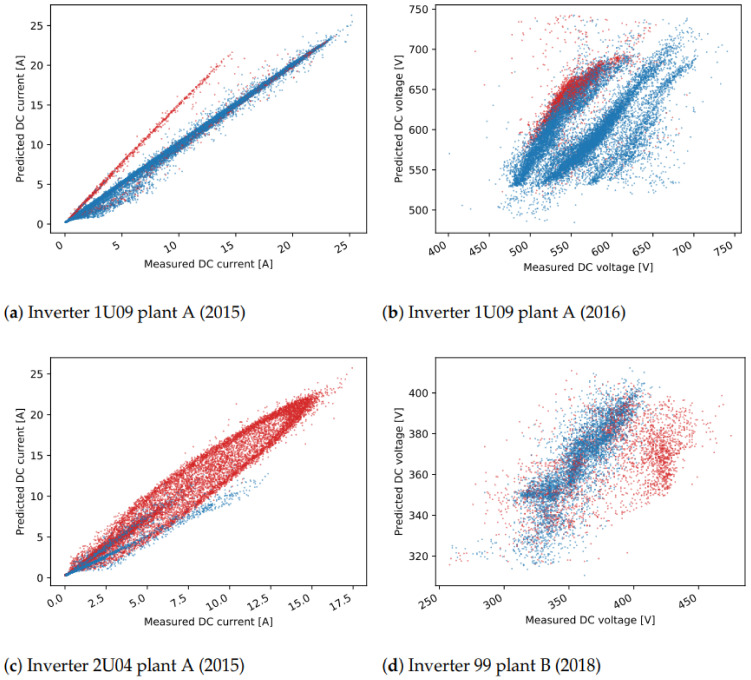
Modeled vs. Predicted values, where the points for the values in the days detected as unusual events are in red.

For many of the detected one-day events, the relative distance to the nearest cluster is less than 50%. In these cases, the residuals show only a slight deviation with respect to the cluster centers (Figure 13b). On the other hand, events with a higher distance usually represent more severe issues. Several events with high distances are detected in December in multiple years, for inverter 2U03 (Figure 12d), which are related to short-term increase or decrease in voltage in several hours in the afternoon. In another example, current-related events are detected for inverter 99, where the current measured for short periods in the afternoon has lower values (Figure 15).

In some cases, events are detected in multiple days over a longer period of time. One such case is seen in mid-2015 (Figure 12a) for inverter 1U09. A scatter plot of the measured values for DC current and the predicted values with the ML model is shown in Figure 15a. The points in red show the values in the days detected as unusual events, where the measured values are lower than the predicted ones for about 5A. Similar scenarios are seen in mid-2016, and in most of the time in 2019 and 2020. The events seen in DC voltage in 2016 for the same inverter are caused by a slight increase in voltage in part or the whole day (Figure 15b). Lower current is also behind the events in 2020 for inverter 1U02, the events in 2019 for 1U03 and 2U01, and finally the events in 2015 for 2U04 (Figure 12). The dependency of the measured and predicted values in the case of inverter 2U04 is not linear, since the orientation of the modules is different than the orientation in inverter 1U01 (Figure 15c). Most of the events detected in DC voltage daily residuals are caused by the high drop in voltage at approximately 19:00 for a short period of time (Figure 12d). On the other hand, for inverter 2U08, the events in 2019 are related to an unusual increase in voltage seen in the morning.

The third scenario, where events in several inverters at the same time are seen, can be observed in a few examples, and they are a probable indication of an anomaly on the reference inverters. In one case, a deviation of the DC current of the reference inverter caused detection of events for all other inverters in plant C in 2017 (Figure 14a). In another case, this time not indicating anomalies on the reference inverter but rather problems in the data collection, the DC current for all inverters in plant A goes to 0 at some times of the day, but also many missing data within the day are seen in the mid-2019 (Figure 12).

Finally, one example of the fourth scenario can be seen for inverter 99 in plant B. In the first half of 2018, for the days detected as events for both DC current and DC voltage, lower DC current and higher DC voltage is observed. The measured and predicted DC voltage in 2018 is seen in Figure 15d. The events of DC current residuals of the inverters 757 and 750 in plant C, detected in 2012 are connected to a drop of current to 0, while at the same time the measured voltage is higher (Figure 14). A similar scenario is detected for inverters 2U01-2U10 in the periods of 8th–12th August 2016 and 25th July–10th August in 2017 (Figure 12).

Overall, the analysis of the detected events shows a successful performance of the method to grasp many truly unusual patterns, especially in the cases where a high distance to the closest cluster is obtained (more than 50%). One limitation is that some events are detected in cases where only a slight deviation from the clusters exists. The sensitivity of the distance metric should be further investigated, and if necessary a different metric could be proposed in future work. Another limitation is that, for online monitoring, if a method for inspecting if the reference inverter itself is operating normally is not implemented, the interpretation of the events should be done with special care.

#### 3.2.3. Control Chart

**Figure 16:**
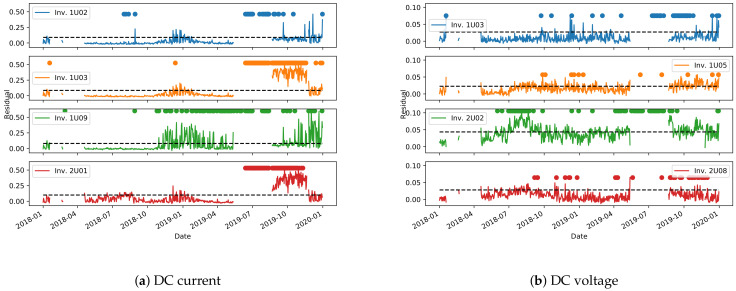
Control charts for anomaly detection on selected inverters from plant A. The dashed horizontal lines are the control chart limits, while the circles are the points in which the clustering algorithms detects an anomaly. Some time periods are not shown in the control chart due to missing data.

**Figure 17:**
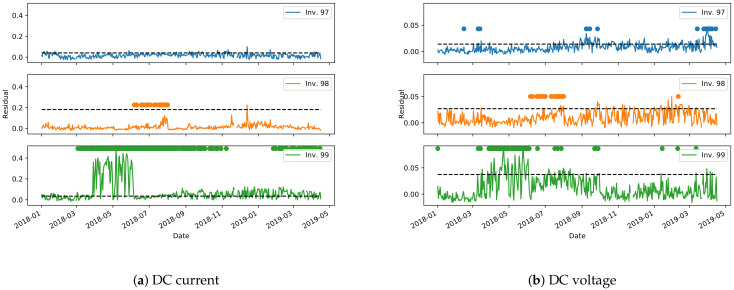
Control charts for anomaly detection on selected inverters from plant B. The dashed horizontal lines are the control chart limits, while the circles are the points in which the clustering algorithms detects an anomaly.

The second method for anomaly detection presented here makes use of the environmental model (Section 2.2.1) to build a control chart on the test set. The model’s performance, as assessed in Section 3.2.1, can be highly variable depending on the plant and the inverter, and therefore the limits for the control chart (Equation (2)) need to be derived on a per-inverter basis. Figure 16 and Figure 17 show the derived control charts for DC current and voltage on the most representative inverters of plants A and B. The dashed horizontal line is the limit defined by Equation (2), while the dots are the points in which the clustering approach from Section 3.2.2 detects an anomaly. Unfortunately, due to missing data, a non-negligible time period is unavailable to derive the control chart for plant A. It can be noted, however, that the two methods for anomaly detection have a good agreement in identifying long periods of anomalous behavior. More localized anomaly peaks, in one method or in the other, are instead most probably outliers, that need to be filtered out.

## 4. Conclusions

In this work, we have presented different data-driven approaches for the assessment of performance degradation in PV plants due to various conditions. The approaches target different data availability and operating conditions, showing a substantial agreement when a comparison is possible. Such methods can be extremely valuable for an efficient operation of a photovoltaic plant, allowing the prompt identification and correction of problems affecting the performance. We have shown that the great degree of variability on PV plants does not affect negatively the accuracy of the algorithms, provided that data of sufficient quality are available for the training phase. Our methods have been validated against some of the most popular methods in the literature, showing comparable performance. Our approaches, however, being data-driven, have the advantage of requiring neither in-depth knowledge of the plant nor specific and accurate physical measurements on-site, rather only the monitoring of the plant with high-level sensors for an adequate amount of time.

The next logical step with respect to anomaly detection would be to allow not just the identification of a failure, but also its characterization in terms of root causes. This, however, would require the collection of much more detailed datasets, where examples of many different kinds of failures would need to be recorded and manually characterized. Our results on the degradation estimation pave also the way for the derivation of predictive models, which can estimate the remaining useful life for all components before the need of replacement due to an unacceptable decrease in performance. Furthermore, for this application, though, the need for datasets with more specific and accurate information about each inverter is mandatory. These considerations reiterate the need for promoting the acquisition of increasingly accurate and detailed datasets monitoring the operation of photovoltaic plants.

## Figures and Tables

**Table 1:** Yearly degradations for plant plant A.

	From PR	From NOCT Power		From Sampled Values	
	Relative deg.	Absolute deg.	Relative deg.	Absolute deg.	Relative deg.
Inverter	[%/Year]	[Wp/Year]	[%/Year]	[W/Year]	[%/Year]
1U01	2.27	490	3.5	390	2.6
1U02	3.91	700	5.1	640	4.4
1U03	2.93	370	2.7	320	2.2
1U04	2.86	300	2.5	560	4.0
1U05	1.83	410	3.0	330	2.3
1U06	2.16	320	2.4	410	2.8
1U07	1.90	180	1.4	350	2.4
1U08	2.17	330	2.5	410	2.9
1U09	2.16	260	2.2	290	2.3
2U01	2.90	200	1.5	310	2.1
2U02	3.26	360	3.0	560	4.0
2U03	1.23	200	1.6	260	1.8
2U04	1.26	430	3.2	230	1.6
2U05	1.84	520	3.8	340	2.3
2U06	1.39	410	3.1	280	1.9
2U07	1.60	500	3.7	280	1.9
2U08	1.18	350	2.7	220	1.5
2U09	1.28	460	3.5	250	1.7
2U10	2.19	640	4.7	390	2.7

**Table 2:** Yearly degradations for plant C.

	From PR	From NOCT/STC Power			From Sampled Values	
	Relative deg.		Absolute deg.	Relative deg.	Absolute deg.	Relative deg.
Inverter	[%/Year]	Conditions	[Wp/Year]	[%/Year]	[W/Year]	[%/Year]
244	0.30	NOCT	14	0.19	29	0.29
		STC	50	0.49		
248	0.61	NOCT	40	0.54	61	0.61
		STC	96	0.97		
242	0.64	NOCT	44	0.59	59	0.58
		STC	85	0.85		
757	0.43	NOCT	18	0.25	37	0.36
		STC	54	0.54		
750	0.75	NOCT	61	0.8	65	0.64
		STC	76	0.75		

**Table 3:** DC current and DC voltage yearly degradation for plant A using the sampled values—based approach.

Inverter	DC Current	DC Voltage	Inverter	DC Current	DC Voltage
	deg. [%/Year]	deg. [%/Year]		deg. [%/Year]	deg. [%/Year]
1U01	1.4	0.46	2U01	1.4	−0.07
1U02	1.5	1.7	2U02	1.4	1.9
1U03	1.4	0.22	2U03	1.2	−0.05
1U04	1.5	0.71	2U04	0.88	−0.02
1U05	1.4	0.14	2U05	1.0	0.11
1U06	1.5	0.34	2U06	0.71	0.19
1U07	1.3	−0.16	2U07	1.1	−0.31
1U08	1.4	0.56	2U08	0.83	−0.26
1U09	1.1	3.6	2U09	1.2	−0.31
			2U10	1.2	0.39

**Table 4:** Average root mean square error (RMSE) of the evaluation data of all prediction models trained to output DC voltage using different models and different input data (without or with the added data).

Algorithm	LR		RFR		SVR		DT	
**Added Data**	**False**	**True**	**False**	**True**	**False**	**True**	**False**	**True**
Plant A	26.2	25.7	20.3	17.3	20.6	15.5	21.7	15.9
Plant B	9.8	9.4	9.1	7.42	9.2	6.99	9.1	7.2
Plant C	9.9	9.9	9.7	9.5	9.8	9.2	9.8	9.7

## Data Availability

The data are not publicly available due to data protection reasons.
